# Influence of storage conditions of small volumes of blood on immune transcriptomic profiles

**DOI:** 10.1186/s13104-020-04980-z

**Published:** 2020-03-13

**Authors:** Rebecca Mathew, Mohammed Toufiq, Valentina Mattei, Muna Al Hashmi, Harshitha Shobha Manjunath, Basirudeen Syed Ahamed Kabeer, Rita Calzone, Chiara Cugno, Damien Chaussabel, Sara Deola, Sara Tomei

**Affiliations:** 1 Omics Core, Research Branch, Out Patient Clinic, Sidra Medicine, PO 26999, Doha, Qatar; 2 System Biology, Research Branch, Out Patient Clinic, Sidra Medicine, Doha, Qatar; 3 Advanced Cell Therapy Core, Sidra Medicine, Doha, Qatar

**Keywords:** Storage, Blood, Trancriptome, QPCR, Immune profiling, Tempus, GenTegra

## Abstract

**Objective:**

Transcriptome analysis of human whole blood is used to discover biomarkers of diseases and to assess phenotypic traits. Here we have collected small volumes of blood in Tempus solution and tested whether different storage conditions have an impact on transcriptomic profiling. Fifty µl of blood were collected in 100µl of Tempus solutions, freezed at − 20 °C for 1 day and eventually thawed, stored and processed under five different conditions: (i) − 20 °C for 1 week; (ii) +4 °C for 1 week; (iii) room temperature for 1 week; (iv) room temperature for 1 day, − 20 °C for 1 day, room temperature until testing at day 7, (v) − 20 °C for 1 week, RNA was isolated and stored in GenTegra solution. We used 272 immune transcript specific assays to test the expression profiling using qPCR based Fluidigm BioMark HD dynamic array.

**Results:**

RNA yield ranged between 0.17 and 1.39µg. Except for one sample, RIN values were > 7. Using Principal Component Analysis, we saw that the storage conditions did not drive sample distribution. The condition that showed larger variability was the RT-FR-RT (room temperature–freezing–room temperature), suggesting that freezing–thawing cycles may have a worse effect on data reproducibility than keeping the samples at room temperature.

## Introduction

The genomic revolution of the last decade and the parallel increase of international collaborations have led to an unprecedented need for transferring biological samples across institutes worldwide. Emerging technologies offer the opportunity to transfer samples at room temperature with the advantage of reducing the logistic challenges associated with sample shipment as well as the carbon footprint associated with portable freezers [1].

Projects aiming at biomarker discovery often employ transcriptional profiling of whole blood [2–5]. Peripheral blood is perhaps the most practical tissue to profile gene expression of the human immune system due to its accessibility, allowing large-scale and non-invasive sampling [6, 7]. Recently, finger-stick blood collection systems have allowed a less invasive and quicker collection of peripheral blood that does not necessarily require medical infrastructures [8–11]. Small volumes of blood are more prone to thaw when compared to blood collected by venipuncture. Often, samples collected in a given place need to be transferred to a second place before processing. Blood samples are generally freezed for storage or shipment to other institutes. Fluctuations in temperature have a high impact on sample performance [12] and suboptimal storage conditions may lead to cell damage [13–15]. When performing gene expression analyses, obtaining high-quality, intact RNA is the most critical step. Due to the ex vivo instability of RNA transcripts [16–19], a crucial challenge is rapid sample handling and mRNA stabilization [7]. There are commercially available tools to assist the investigators in preserving RNA integrity prior to downstream analyses [20].

In this study we have decided to use the Tempus system (ThermoFisher Scientific) as its performance has been showed to be greater than other systems by previous investigation [21]. When blood is drawn into Tempus Blood RNA tubes and mixed, the stabilizing reagents lyse cells almost immediately. At the same time, cellular RNases are inactivated and RNA is selectively precipitated, leaving genomic DNA and proteins in solution. For RNA isolation, the Tempus system uses a solid-phase, silica-based purification strategy. RNA integrity should also be ensured post-RNA isolation. RNA is generally stored at − 80 °C. Nevertheless, emerging technologies are now offering the opportunity to keep the samples at room temperature, such as GenTegra (GenVault, Carlsbad, CA). GenTegra is an inorganic matrix with oxidation protection and antimicrobial activity for storage of isolated RNA at room temperature. It is supplied as transparent coating at the bottom of the GenTegra RNA tube. Purified RNA is added to GenTegra tube, dried down and eventually recovered with the addition of water.

**Fig. 1:**
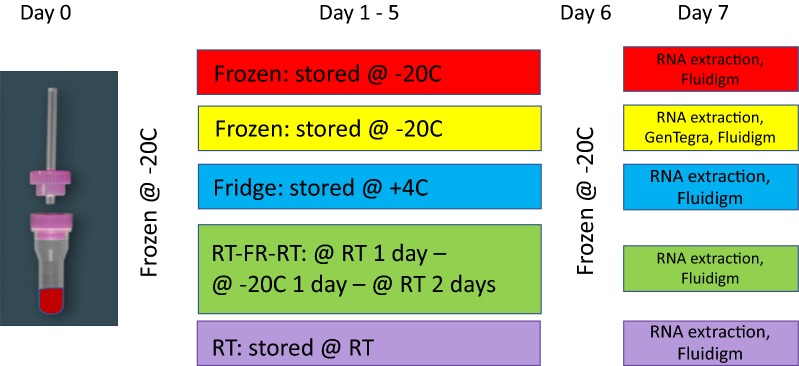
Workflow of the storage conditions. *RT* Room Temperature, *FR* Freezing

The aim of this study was (1) to compare different storage conditions on small volumes of blood and to assess the effect of GenTegra storage solution with regard to quality/quantity of RNA (Fig. 1 displays the experiment rationale) and (2) to evaluate whether expression profiling is affected by the different storage conditions.

## Main text

### Sample collection

Blood samples were collected as previously described [10]. Ethical approvals were collected from Sidra Institutional Review Board committee (IRB Protocol #1707011887). Three samples from an immune competent subject were collected for each storage condition. Samples were processed for downstream qPCR analysis in duplicates. Fifty µl of blood were collected in 100 µl of Tempus solutions and stored according to the diagram in Fig. 1. Tempus Spin RNA Isolation kit (ThermoFisher, Waltham, Massachusetts, USA) was used to isolate and purify RNA from blood collected in the capillary tubes according to the manufacturer’s instructions. After extraction, RNA quality and quantity were checked on Bioanalyzer (Agilent Technologies, Carlsbad, California, US). Samples were evaluated according to their RIN (RNA integrity number). This score is classified on a numbering system from 1 to 10, with 1 indicating the most degraded RNA and 10 indicating the most intact RNA.

We also tested the stability of samples after addition of GenTegra RNA solution. Fifty µl of RNA were added to the GenTegra RNA coating and mixed well to ensure homogeneity. Samples were then dried using a vacuum concentrator according to GenTegra RNA standard protocol. The dried samples were kept at room temperature for 1 week prior to testing. For RNA recovery, a volume of molecular-grade water equivalent to the input volume was added to the dried samples. The concentration and integrity of rehydrated RNA were checked. RNA was then processed for the downstream applications.

### Fluidigm gene expression

Gene expression was performed by parallel quantitative PCR using the high-throughput BioMark HD platform (Fluidigm Co., San Francisco, CA, USA), according to the manufacturer’s instructions. Transcripts were selected according to previous literature [22]. Eight genes were used as housekeeping, namely: DOCK2, EEF1A1, FAM105B, FTL, MYL6, MYL12B, RPS10, and RPS25. A good quality RNA was also used as internal reference. Transcript specific assays (DeltaGene Assays) were designed and ordered through D3 Assay Design (https://d3.fluidigm.com/account/login). Reverse transcription was performed on the isolated RNA using Fluidigm cDNA synthesis kit (Fluidigm Co., San Francisco, CA, USA). The PCR reaction conditions were set following the Biomark HD Protocol GE 96 × 96 Fast PCR + Melt v2. Each PCR reaction used distilled water instead of cDNA as negative control. Melting curves were generated for each gene. Samples were run in duplicates.

QPCR analysis Ct values (expression values) were exported from Fluidigm Real-Time PCR Analysis Software as *csv file and processed using Partek Genomic Suite version 7.18 (Partek, Chesterfield, Missouri, USA). Genes corresponding to each sample with expression beyond the detectable range were set as missing values for further analysis. Technical replicates of each sample were averaged. The geometric mean of the eight housekeeping genes was subtracted from the Ct values of each sample to give a Delta Ct value that corrects for different sample amounts. Delta Ct values were transformed to the negative Delta Ct values prior to performing Principal Component Analysis. ggplot2 library (version 3.5.2) in R was used to generate Boxplot for the eight housekeeping genes and immune-related genes across different storage conditions.

### Data analysis

Analysis of variance (ANOVA) was applied to compare data from the different storage conditions. Principal component analysis (PCA) was applied for visualization when relevant. Non-parametric Wilcoxon tests were used to evaluate statistical differences between the storage conditions. All statistical tests were two-sided. *P*-values lower than 0.05 were considered statistically significant.

### Results

#### RNA quality and yield

**Fig. 2:**
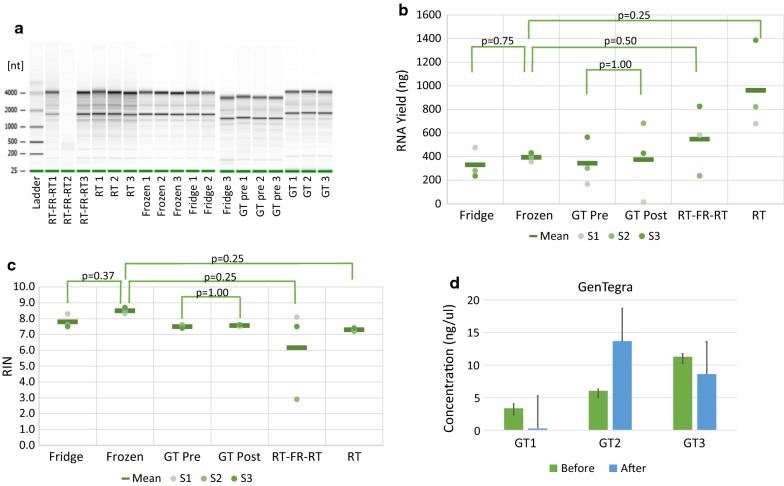
Bioanalyzer profiles of the RNA samples from the different storage conditions. *RT* Room Temperature, *FR* Freezing, *GT* GenTegra (**a**). Comparison of the RNA yield across the different storage conditions (**b**). Comparison of RIN values across the different storage conditions (**c**). Comparison of RNA concentration of GenTegra samples pre- and post-treatment (**d**)

Figure 2a shows the Bioanalyzer profiles of the RNA samples according to the storage conditions. The Bioanalyzer run gave a RIN score > 7 for all the samples except sample “RT-FR-RT2” (RIN 2.9). RNA yield ranged between 0.17 and 1.39 µg for the non-GenTegra treated samples (Additional file 1: Table S1). One sample treated with GenTegra had a yield of 15 ng. Further studies employing a higher number of samples are required to confirm our findings. The RNA yield was overall higher in the “RT” condition, followed by “RT-FR-RT” condition. However no significant difference was found (Fig. 2b). RIN values were comparable across the different storage conditions, except for one sample of the “RT-FR-RT” condition that displayed a RIN value of 2.9 (Fig. 2c). The “Frozen” condition showed the highest mean RIN value, however no significant difference was found when assessing RIN values across conditions, suggesting that the different storage conditions did not have a significant impact on either yield or RIN values.

Even if the concentration of one sample treated with GenTegra was lower as compared to the other two (Fig. 2d), our results demonstrate that overall the RNA stored at room temperature with GenTegra technology performed equivalently to the other samples tested. The workflow for GenTegra was straightforward, involving the addition of RNA to the product tubes, drying, storage at room temperature for 1 week and recovery with water (the volume was kept equal to the initial volume). No additional clean up or purification was required after rehydration. The RNA concentration did not differ significantly after the recovery of RNA (Fig. 2d, Wilcoxon test on “Before” vs “After” samples, *p-*value 1.0). Most importantly, the GenTegra RNA coating did not appear to interfere with any of the downstream applications, although it did affect the A260/A280 and A260/A230 ratio but not significantly (Additional file 1: Table S2). This has also been reported in another study aiming at assessing the stability of DNA using GenTegra technology [1].

#### Immune related transcriptional profiling across the conditions

**Fig. 3:**
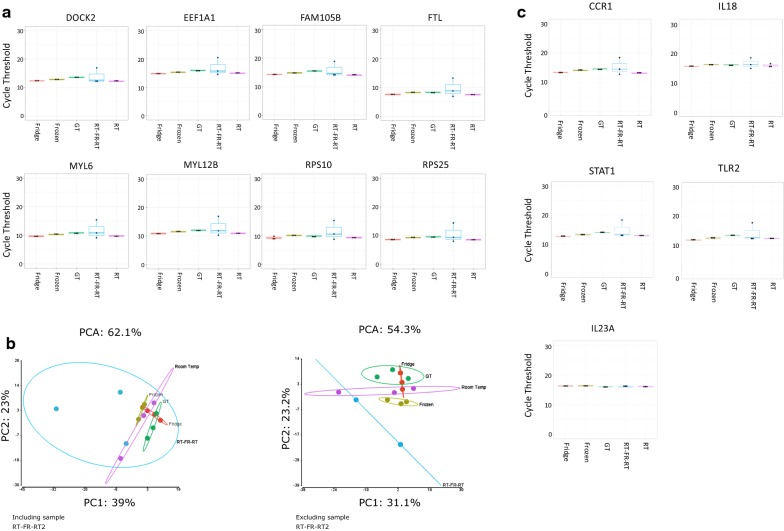
**a** Box and whisker plots of Ct of the eight housekeeping genes across the different storage conditions. **b** Principal component analysis of the different storage conditions on the complete dataset, including and excluding the poor quality sample RT-FR-RT2. *RT* room temperature, *FR* Freezing, *GT* GenTegra. **c** Box and whisker plots of five immune-related genes across the different storage conditions

We next sought to determine whether any transcriptional differences existed between the different storage conditions. We employed Fluidigm-based transcriptome profiling for immune-related genes as previously described [22]. We expected to see transcriptional similarity across the different conditions. As expected, by plotting Ct values of the eight housekeeping genes, we found no significant differences across the storage conditions, suggesting that the different storage conditions did not impact transcriptome profiling (Fig. 3a). Nevertheless, the condition “RT-FR-RT” showed higher variability across the replicates, suggesting that freezing–thawing cycles may increase transcriptional variance even in the presence of the Tempus stabilizer solution, and should be avoided in studies aiming at immune-related transcriptional profiling. In a second step, we performed principal component analysis (PCA) on the complete transcriptional data set visualizing the three-dimensional distribution of the samples according to their storage conditions. The assignment of the individual samples to the five different storage conditions did not predict clearly their distribution in a three-dimensional space although samples belonging to the same conditions tended to cluster together; this suggested that the different storage conditions did not have a different impact on transcriptomic profiling (Fig. 3b). We next selected immune-related genes and assessed differences in their Ct values across the different storage conditions. These genes included: IL18, IL23A, STAT1, TLR2 and CCR1. As expected, we found no significant differences, suggesting that the different storage conditions did not alter the transcriptomic profiles of the selected immune-related genes (Fig. 3c, Additional file 1: Table S3).

### Discussion

The quality of gene expression data is strictly dependent on the integrity and stability of the mRNA [20]. This, in turn, is related to the proper optimal storage and processing of the samples as well as on downstream technologies used [16, 23]. Maintaining consistent storage conditions is critical for data reproducibility as even native specimens of peripheral blood may undergo significant changes of gene expression patterns due to gene induction, repression and RNA degradation, if not properly handled [24]. Suboptimal freezing and thawing conditions lead to cell damage by two basic mechanisms: (i) the mechanical damage caused by the formation of intracellular ice crystals [25, 26], and (ii) the osmotic damage due to high intracellular salt concentrations as a result of water loss [27]. Preventing cell damage mechanisms during sample collection, storage and processing is of paramount importance in any experimental setting.

MRNA stabilization is essential for gene expression analyses and is required for multicenter clinical and research programs.

Here we chose Tempus RNA solution as stabilizing reagent as several study have previously shown a greater performance of RNA Tempus solution when compared to other stabilizing reagents, in terms of RNA quantity and quality [28–30]. We tested whether different storage conditions had an impact on transcriptomic profiling of small volumes of peripheral blood collected in Tempus stabilizer solution.

Previous studies have demonstrated that changes in gene expression can be due to various factors, including glucose depletion, changes in pH, lactate accumulation, hypoxia etc., causing biological stresses [7, 31]. Nevertheless, to the best of our knowledge, there are no studies assessing the effect of storage conditions on small volume of peripheral blood collected in Tempus stabilizer solution.

No statistical difference was found when comparing RNA yield and RIN values across the different conditions. All samples from the different conditions had a RIN value above 7 except for one sample in the “RT-FR-RT” group that had a RIN value of 2.9. The overall quality was considered sufficient as standard RIN above 5 was set as a cut-off for downstream applications including microarray and qPCR [16, 32]. No significant difference was found when assessing immune-related transcriptomic profiles across the different storage conditions, suggesting Tempus stabilizer as an effective solution to ensure sample performance even if handed at different storage conditions.

Our experiments demonstrated that the different storage conditions were able to maintain RNA integrity with no loss of sample quality in downstream applications such as Fluidigm-based gene expression testing. These results offer opportunities for collaborative institutions to choose the storage conditions for peripheral blood collected in Tempus tubes most suitable to their needs making sure to avoid freezing–thawing cycles.

## Limitations

- The study was conducted on a limited number of samples.
- The PCR experiments were performed on a selected gene panel rather than the transcriptome at the global level.

## Supplementary information

**Additional file 1: Table S1.** Yield (ng) of the three replicates of the following conditions: RT-FR-RT, RT, Frozen and Fridge. **Table S2.** Nanodrop A260/A280 and A260/A230 values before and after the addition of GenTegra (Wilcoxon test, A260/A280 p-value: 0.25; A260/A230 p value: 1.00). **Table S3**. Multi-comparison tests across the different storage conditions for selected immune-related genes and housekeeping genes.

## Data Availability

The datasets used and/or analysed during the current study are available from the corresponding author on reasonable request.

## Abbreviations

ANOVA: Analysis of Variance
PCA: Principal component analysis
qPCR: Quantitative polymerase chain reaction
RIN: RNA integrity number
