# Supplementary material for: Influence of storage conditions of small volumes of blood on immune transcriptomic profiles
Source: BMC Res Notes. 2020 Mar 13;13:150. doi: 10.1186/s13104-020-04980-z (PMC7069204; doi:10.1186/s13104-020-04980-z)
Supplement: Supplementary file 1 — Additional file 1: Table S1. Yield (ng) of the three replicates of the following conditions: RT-FR-RT, RT, Frozen and Fridge. Table S2. Nanodrop A260/A280 and A260/A230 values before and after the addition of GenTegra (Wilcoxon test, A260/A280 p-value: 0.25; A260/A230 p value: 1.00). Table S3. Multi-comparison tests across the different storage conditions for selected immune-related genes and housekeeping genes. [file 13104_2020_4980_MOESM1_ESM.docx]

**Additional file 1: Table S1.** Yield (ng) of the three replicates of the following conditions: RT-FR-RT, RT, Frozen and Fridge.

| **Sample ID** | **Yield (ng)** | **RIN** |
| --- | --- | --- |
| RT-FR-RT 1 | 584 | 8.1 |
| RT-FR-RT 2 | 237 | 2.9 |
| RT-FR-RT 3 | 826 | 7.5 |
| RT 1 | 679 | 7.3 |
| RT 2 | 822 | 7.2 |
| RT 3 | 1386 | 7.4 |
| Frozen1 | 357 | 8.3 |
| Frozen 2 | 395 | 8.5 |
| Frozen 3 | 431 | 8.7 |
| Fridge 1 | 477 | 8.3 |
| Fridge 2 | 281 | 7.6 |
| Fridge 3 | 237 | 7.5 |

**Additional file 1: Table S2.** Nanodrop A260/A280 and A260/A230 values before and after the addition of GenTegra (Wilcoxon test, A260/A280 p-value: 0.25; A260/A230 p value: 1.00).

| **Sample ID** | **Yield (ng)** | | | **A260/A280** | | **A260/A230** | |
| --- | --- | --- | --- | --- | --- | --- | --- |
|  | Before | After | Before | | After | Before | After |
| **GT1** | 167 | 15 | 1.85 | | 0.34 | 1.23 | 0.01 |
| **GT2** | 302 | 682 | 1.82 | | 1.32 | 1.30 | 3.76 |
| **GT3** | 564 | 428 | 1.80 | | 1.39 | 1.09 | 0.81 |

**Additional file 1: Table S3**. Multi-comparison tests across the different storage conditions for selected immune-related genes and housekeeping genes.

| **Gene** | **Conditions** | **P-value** |
| --- | --- | --- |
| IL18 | Frozen vs Fridge | 0.1979 |
|  | Frozen vs GT | 0.7864 |
|  | Frozen vs RT-FR-RT | 0.9837 |
|  | Frozen vs RT | 0.7766 |
| IL23A | Frozen vs Fridge | 0.9975 |
|  | Frozen vs GT | 0.2984 |
|  | Frozen vs RT-FR-RT | 0.4659 |
|  | Frozen vs RT | 0.6470 |
| STAT1 | Frozen vs Fridge | 0.3387 |
|  | Frozen vs GT | 0.1698 |
|  | Frozen vs RT-FR-RT | 0.7705 |
|  | Frozen vs RT | 0.4504 |
| TLR2 | Frozen vs Fridge | 0.3608 |
|  | Frozen vs GT | 0.2222 |
|  | Frozen vs RT-FR-RT | 0.7435 |
|  | Frozen vs RT | 0.8246 |
| CCR1 | Frozen vs Fridge | 0.2025 |
|  | Frozen vs GT | 0.1864 |
|  | Frozen vs RT-FR-RT | 0.8794 |
|  | Frozen vs RT | 0.0532 |
| all housekeeping genes | Frozen vs Fridge | 0.3836 |
|  | Frozen vs GT | 0.3500 |
|  | Frozen vs RT-FR-RT | 0.8407 |
|  | Frozen vs RT | 0.0816 |
